# The safety, tolerability, pharmacokinetics, and pharmacodynamics of nebulized pegylated interferon α-2b in healthy adults: a randomized phase 1 trial

**DOI:** 10.1186/s40360-025-00937-9

**Published:** 2025-05-12

**Authors:** Wenqi Huang, Yanneng Kang, Yajun Zhao, Jiao Yang, Changjuan Dai, Weibing Wu, Jinchao Xu, Wen Jin, Xiaolu Wu, Qing Zhou

**Affiliations:** Department of Hepatology, Xiamen Humanity Hospital Fujian Medical University, Xiamen, Fujian 361000 China

**Keywords:** Pegylated interferon α-2b, Nebulization, Safety, Pharmacodynamics

## Abstract

**Background:**

Interferons (IFNs) are proteins that combat viruses and regulate the immune system. Studies have demonstrated that aerosol inhalation of IFNα is both effective and safe for treating respiratory infections. However, IFNα has a short half-life and is rapidly cleared by lung defenses. Polyethylene glycol (PEG) ylation is a common strategy to extend the duration of drug action. PegIFNα-2b is a long-acting interferon formed by the covalent binding of 40 kDa Y-shaped branched PEG with recombinant human IFNα-2b. This study aimed to assess the safety, tolerability, pharmacokinetic, and pharmacodynamic characteristics of nebulized PegIFNα-2b in healthy adult subjects, providing guidance for further clinical investigations.

**Methods:**

This study employed a randomized, controlled clinical trial design with a total of 18 healthy adult subjects enrolled. Participants were randomly assigned in a 1:1:1 ratio to three groups. Treatment group 1 and group 2 received 90 µg and 180 µg of nebulized PegIFNα-2b, respectively, while the control group was administered a combination of 180 µg PegIFNα-2b and 15 mg inhalable Ambroxol Hydrochloride solution, all in a single dose. Safety, tolerability, and blood drug concentration were assessed, along with blood neopterin levels for pharmacokinetic and pharmacodynamic evaluation.

**Results:**

The incidence of adverse events (AEs) was 38.9% (7/18) with no significant difference among the groups (*P* > 0.05). AEs included anemia (*N* = 5) and leukopenia (*N* = 2), predominantly of grade 1 severity (6/7), with no severe events. Blood PegIFNα-2b concentrations were below detection limits in most subjects, except one in treatment group 2. Neopterin levels were generally low in treatment group 1 and the control group, with slightly higher observed in most subjects of treatment group 2, but differences were not significant (*P* > 0.05).

**Conclusions:**

Nebulized PegIFNα-2b at doses of 90 µg and 180 µg showed acceptable safety and tolerability. Minimal systemic absorption was observed following inhalation. Further studies are needed to explore its potential, especially in patients with lower respiratory tract infections.

**Clinical trial registration:**

ChiCTR2300074909, retrospectively registered in https://www.chictr.org.cn/ at 20 August 2023.

**Supplementary Information:**

The online version contains supplementary material available at 10.1186/s40360-025-00937-9.

## Introduction

Acute lower respiratory tract infections continue to pose a significant and widespread challenge to public health, imposing a heavier global disease burden compared to human immunodeficiency virus (HIV) infection, malaria, cancer, or myocardial infarction [1]. Viruses play a pivotal role in respiratory tract infections, with rhinoviruses, influenza viruses, coronaviruses (CoVs), respiratory syncytial virus (RSV), human metapneumovirus (hMPV), and parainfluenza viruses (PiVs) being the primary offenders. Nevertheless, despite the availability of targeted antiviral treatments for influenza viruses and cytomegalovirus, supportive care and symptomatic management remain the primary treatment options for infections caused by other viruses [2].

Interferons (IFNs) are a class of cytokines with broad-spectrum antiviral and immunomodulatory effects. They induce a state of resistance against pathogens in infected cells, activate the innate immune system, trigger adaptive immune responses, and promote immunological memory, thereby playing a pivotal role in antiviral defense mechanisms [3]. Most IFNs medications are administered via subcutaneous or intramuscular injection, which may lead to systemic adverse reactions such as flu-like symptoms, resulting in poor patient compliance. In recent years, abundant clinical experience in pediatric medicine and multicenter research data have suggested that nebulized inhalation of IFNα, as a localized treatment approach, can improve symptoms related to lower respiratory tract infections, shorten hospital stays, and demonstrate good safety [4–6]. However, conventional IFNα, as a protein drug, faces challenges in local administration, such as a short half-life, clearance by pulmonary ciliary mucosa, phagocytosis by alveolar macrophages, and enzymatic metabolism, all of which reduce its effective duration of action [7]. For drugs intended for local pulmonary action, both the duration of lung residence and the local drug concentration directly determine their efficacy in the lungs. Polyethylene glycol (PEG)ylation is the most common strategy for prolonging the residence of drugs in the lungs. Compared to conventional IFNα, PEGylated IFNα (PegIFNα) exhibits a 2.5- to 3-fold increase in lung residence [8, 9]. Previous studies in the treatment of chronic hepatitis B have demonstrated that PegIFNα has significantly superior antiviral activity compared to conventional IFNα [10, 11]. Therefore, considering its drug characteristics, administration environment, and previous antiviral research, nebulized inhalation of PegIFNα may further enhance the therapeutic efficacy of lower respiratory tract infections. However, there is currently no related research available.

PegIFNα-2b (Pegbing^®^, Xiamen Amoytop Biotech Co., Ltd.) is a long-acting interferon formed by the covalent binding of 40 kDa Y-shaped branched PEG with recombinant human IFNα-2b [12]. It was approved in September 2016 for subcutaneous injection in the treatment of chronic hepatitis B and chronic hepatitis C. A study in New Zealand rabbits, using pulmonary liquid quantification nebulization to administer the same dose of PegIFNα-2b or conventional IFNα-2b, showed that lung exposure to the drug at 48 h post-administration was approximately 10 times higher in the PegIFNα-2b group compared to the conventional IFNα-2b group. Furthermore, drug concentration could still be detected in the PegIFNα-2b group at 72 h post-administration, while it was undetectable in the conventional IFNα-2b group (unpublished data). Based on these findings, nebulized inhalation of PegIFNα-2b is expected to reduce dosing frequency, improve patient compliance, increase local drug concentration, and prolong drug residence time, thereby enhancing clinical treatment outcomes.

This phase 1 clinical study aimed to explore the safety and pharmacokinetic/pharmacodynamic characteristics of nebulized inhalation of PegIFNα-2b in healthy individuals, thereby laying the groundwork for subsequent clinical research in patient populations.

## Methods

### Study design

This was a randomized, controlled, single-center phase 1 clinical study conducted in China (ChiCTR2300074909) from August 10, 2023 to August 24, 2023. Participants were randomly allocated in a 1:1:1 ratio to treatment group 1 (nebulized PegIFNα-2b 90 µg/0.5mL), treatment group 2 (nebulized PegIFNα-2b 180 µg/0.5mL), and the control group (nebulized PegIFNα-2b 180 µg/0.5mL combined with Ambroxol Hydrochloride).

The study employed a compression nebulizer (PARI TurboBOY, PARI GmbH, Germany) for aerosolization, with gas flow adjusted to 7 L/min, spray volume set at 0.4 mL/min, and spray rate at 0.25 mL/min. All participants used a standardized mouthpiece for drug nebulization. Aerosol particle size distribution was characterized using laser diffraction analysis (Malvern Mastersizer X, Malvern Panalytical Ltd., UK).

The aerosol solution for treatment groups 1 and 2 consisted of 2 mL of 0.9% sodium chloride injection + 0.5 mL of PegIFNα-2b (90–180 µg), while the control group received 2 mL of inhalable Ambroxol Hydrochloride solution (15 mg) + 0.5 mL of PegIFNα-2b (180 µg). All subjects underwent aerosolization once, and safety, tolerability, and pharmacokinetic/pharmacodynamic characteristics were observed and measured over 7 days (at 0.5 h, 1 h, 3 h, 5 h, 8 h, 12 h, 24 h, 48 h, 72 h, 120 h, and 168 h post-administration).

The dosages of PegIFNα-2b used in this study were based on guidelines recommending the dosage of conventional IFNα for nebulized inhalation in children with lower respiratory tract infections as 200,000-400,000 IU/kg/dose [13]. Within this recommended range, children do not experience fever, chills, or other flu-like symptoms. Based on the median birth weight of Chinese children, which is 3.32 kg for boys and 3.21 kg for girls [14], the median dose of nebulized inhalation of IFNα is calculated to be 664,000–1,328,000 IU/dose for boys and 642,000–1,284,000 IU/dose for girls. Therefore, it is anticipated that nebulized inhalation of PegIFNα-2b 90 µg (330,000 IU) or 180 µg (660,000 IU) will demonstrate good safety and tolerability. Preliminary animal studies of PegIFNα-2b (unpublished) have shown that, when administered at the same dosing regimen, PegIFNα-2b remains in the lungs for a longer duration and is metabolized more slowly compared to conventional IFNα-2b. Specifically, conventional IFNα-2b is metabolized within 10 h in the lungs, whereas PegIFNα-2b takes more than 72 h for metabolism. These findings suggest that PegIFNα-2b can be administered at a lower dose compared to conventional IFNα-2b, while still maintaining effective drug delivery and therapeutic action. Furthermore, 180 µg is the maximum approved dose of PegIFNα-2b for human use, with 90 µg and 180 µg being the most commonly used nebulized doses in clinical practice.

During the study period, participants were instructed to follow specific lifestyle guidelines to minimize potential confounding factors and ensure the reliability of the results: (1) Dietary Management: Participants were instructed to refrain from consuming water for 1 h prior to and 2 h following drug administration, and to avoid food intake from 8 h before to 2 h after administration. Throughout the study, participants were advised to avoid grapefruit, citrus fruits, carbonated foods, and beverages containing xanthine (e.g., coffee, tea, chocolate, cola, and fruit juices). Additionally, caffeine, tea, smoking, and alcohol were prohibited. (2) Exercise Management: Participants were advised to refrain from engaging in high-intensity physical activities, such as strength training, aerobic exercise, and sports like football, to minimize the potential impact of exercise on the study outcomes.

The study protocol was approved by the Institutional Review Board/Ethics Committee of the research center and conducted in accordance with the International Council on Harmonization guidelines on Good Clinical Practice, the principles of the Helsinki Declaration, and regulatory requirements in China. All participants provided informed consent.

## Participants

Inclusion criteria were as follows: (1) Age between 18 and 50 years, regardless of gender; (2) Female participants must weigh ≥ 45 kg, male participants must weigh ≥ 50 kg, with a Body Mass Index (BMI) between 18.5 and 28 kg/m^2^; (3) Women of reproductive potential must have a negative pregnancy test result during screening; (4) Participants (including their partners) must voluntarily adopt effective, non-pharmacological contraceptive measures from before administration until 3 months post-administration, and must not have plans for sperm or egg donation; alternatively, participants (including their partners) must be infertile (having undergone surgical sterilization or being in menopause).

The primary exclusion criteria included: (1) Clinically significant abnormalities in physical examination, vital signs, or laboratory tests; (2) Pulmonary function test results: Forced Expiratory Volume in 1 s (FEV1) measured value / FEV1 predicted value ≤ 80% or Forced Vital Capacity (FVC) ≤ 80% of predicted value; (3) History of clinically significant diseases affecting the cardiovascular, hematologic and lymphatic, respiratory, urinary, endocrine, immune, psychiatric or neurological systems (such as epilepsy); (4) History of ocular or thyroid-related diseases deemed clinically significant by the investigator; (5) Alcohol or drug abuse, blood donation, or significant blood loss (> 450 mL) within the 3 months prior to screening; (6) Known or suspected allergies to investigational drugs or excipients.

## Objectives and endpoints

### Primary endpoints

#### Safety and tolerability assessment

Participants were monitored at 0.5 h, 1 h, 3 h, 5 h, 8 h, 12 h, 24 h, 48 h, 72 h, 120 h, and 168 h after nebulization. The follow-up included monitoring of vital signs, physical examination, assessment of serious adverse events (SAEs) and adverse events (AEs), as well as laboratory investigations for safety assessment, including complete blood count, urinalysis, liver function tests, renal function tests, electrocardiography, and chest imaging. All these assessments were conducted by the research center laboratory.

### Secondary endpoints

#### Pharmacokinetic and pharmacodynamic assessment

Blood samples were collected from participants before and after nebulization at multiple intervals—0.5 h, 1 h, 3 h, 5 h, 8 h, 12 h, 24 h, 48 h, 72 h, 120 h, and 168 h post-nebulization for both pharmacokinetic and pharmacodynamic assessments. These samples were sent to the central laboratory for analysis. The pharmacokinetic assessment focused on analyzing drug concentrations, while the pharmacodynamic assessment involved analyzing neopterin levels, as well as monitoring participant temperature and neutrophil counts.

The concentration of PegIFNα-2b was quantitatively measured using the Quantikine™ HS ELISA Human IFN-α2 Immunoassay kit, with a lower limit of quantification (LLOQ) of 3 pg/mL. The concentration of neopterin was detected using LC-MS/MS, with an LLOQ of 0.5 ng/mL.

### Statistical analysis

No statistical hypotheses or methods were used to calculate the sample size. Participants were randomly assigned to three groups in a 1:1:1 ratio using a simple randomization method. Sealed, light-tight envelopes with random numbers on the outside were provided to the researchers.

Safety data were presented in tabular and/or graphical format and summarized descriptively.

Based on the blood drug concentration-time curve, the following pharmacokinetic analysis parameters were determined as allowed by the data: area under the concentration-time curve (AUC_0 − t_, AUC_0−∞_), maximum concentration (C_max_), time to reach maximum concentration (T_max_), half-life (t_1/2_), apparent clearance rate (CL), apparent volume of distribution (Vd), and mean residence time (MRT). Based on the neopterin concentration-time data, the following kinetic parameters were determined if the data permitted: AUC_0 − t_ and C_max_ of neopterin concentration. In addition, trends in the absolute values of neutrophils and body temperature were described.

All participants who were randomized and received at least one dose of the investigational drug were included in the safety analysis. Participants who were randomized, had baseline data, and had at least one post-dose evaluable data point were included in the pharmacokinetic and pharmacodynamic analysis.

Statistical analyses were performed using SAS 9.4 (SAS Institute, Inc., Cary, NC, USA). All statistical tests were two-tailed, and *P* ≤ 0.05 was considered statistically significant (unless otherwise specified).

## Results

### Aerosol particle size measurement results

The mass median aerodynamic diameter (MMAD) was measured to be 3.5 μm, and the fine particle fraction (FPF) for particles less than 5 μm was 67%.

### Participants and baseline characteristics

**Fig. 1:**
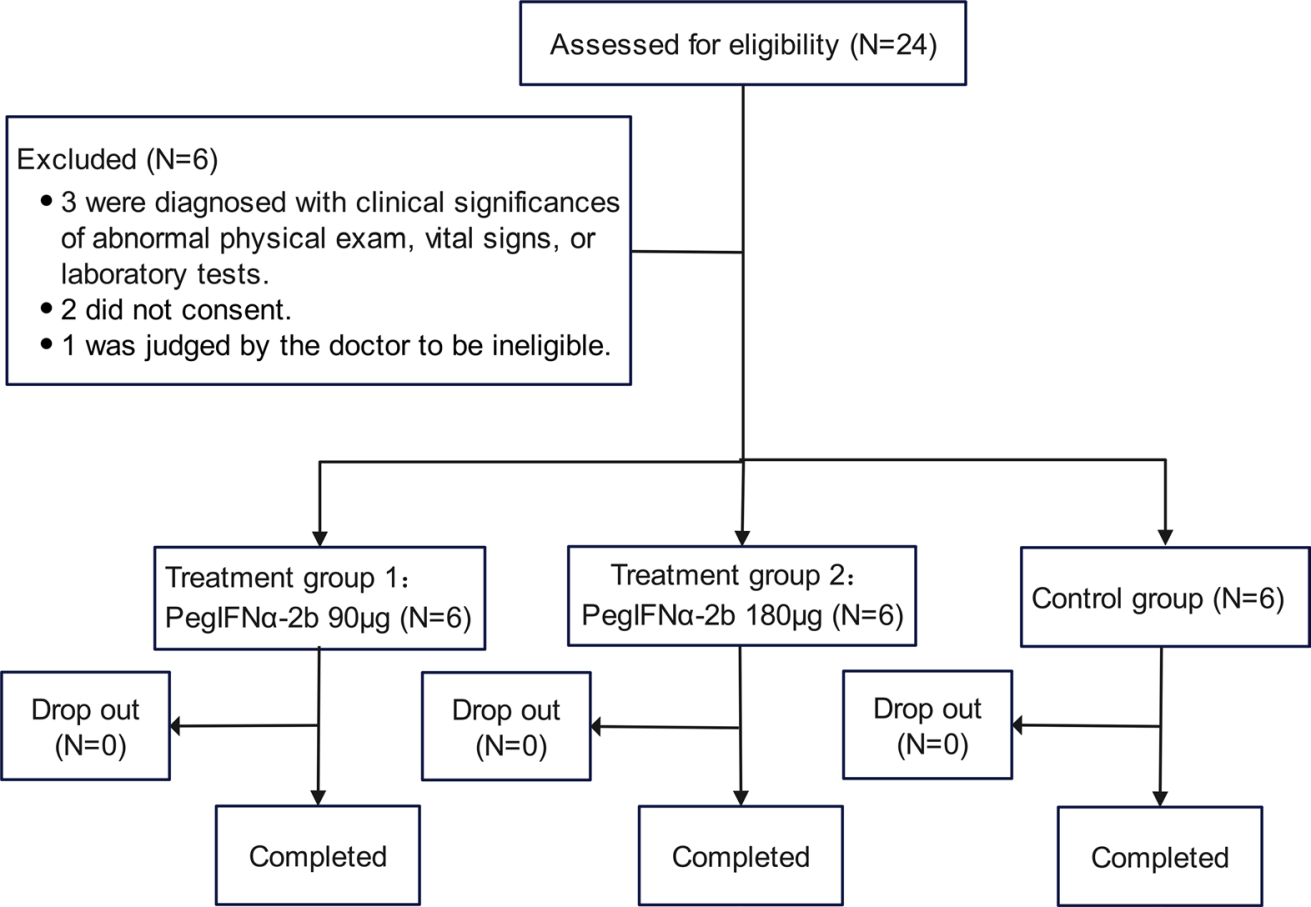
Trial profile

A total of 18 healthy adult participants were enrolled in the study (Fig. 1), with 6 participants each allocated to treatment group 1 (nebulized PegIFNα-2b 90 µg/0.5mL, *n* = 6), treatment group 2 (nebulized PegIFNα-2b 180 µg/0.5 mL, *n* = 6), and the control group (nebulized PegIFNα-2b 180 µg/0.5mL combined with Ambroxol Hydrochloride, *n* = 6). The mean age was 26.8 (± 8.55) years. Demographic characteristics, including height, weight, and other indicators were comparable across the groups (Table 1). All participants completed a single nebulized dose administration.

**Table 1:** Study demographics and baseline characteristics (*n* = 18)

	Treatment group 1 (*n* = 6)	Treatment group 2 (*n* = 6)	Control group (*n* = 6)	Total (*n* = 18)
Age (years), mean (SD)	26.3 (7.61)	25.8 (11.11)	28.2 (7.99)	26.8 (8.55)
Gender, n (%)				
Male	1 (16.7)	2 (33.3)	2 (33.3)	5 (27.8)
Female	5 (83.3)	4 (66.7)	4 (66.7)	13(72.2)
Nationality, n (%)				
Han	5 (83.3)	6 (100.0)	6 (100.0)	17 (94.4)
Other	1 (16.7)	0	0	1 (5.6))
Height (m), mean (SD)	1.6493 (0.0742)	1.6547 (0.0399)	1.6253(0.0717)	1.6431 (0.0614)
Weight (kg), mean (SD)	59.42 (10.004)	58.27 (7.464)	57.78 (10.018)	58.49 (8.708)
Body surface area (m^2^), mean (SD)	21.73 (2.328)	21.23 (2.094)	21.75 (2.469)	21.57 (2.177)
Neutrophil counts (×10^9^/L), mean (SD)	3.087 (0.7109)	3.320 (1.0825)	3.520 (0.8917)	3.309 (0.8720)

### Primary endpoints: Safety and tolerability assessment

During the study period, 7 participants (38.9%) experienced AEs (Table 2), including anemia (5/7) and leukopenia (2/7), which did not require intervention and resolved spontaneously. The severity of anemia was Grade 1 in all cases, beginning with the 5th blood draw on the first day, likely attributed to frequent blood sampling. One case of leukopenia in treatment group 2 was Grade 2, and one case in treatment group 1 was Grade 1. No SAEs occurred, indicating overall good safety and tolerability. There were no significant differences in the incidence of adverse events among the groups (*P* > 0.05).

**Table 2:** Adverse events (*n* = 18)

	Treatment group 1 (*n* = 6)		Treatment group 2 (*n* = 6)		Control group (*n* = 6)		Total (*N* = 18)	
	Cases (*n*, %)	Events	Cases (*n*, %)	Events	Cases (*n*, %)	Events	Cases (*n*, %)	Events
All events	4 (66.7)	4	1 (16.7)	1	2 (33.3)	2	7 (38.9)	7
Serious events	0 (0)	0	0 (0)	0	0 (0)	0	0 (0)	0
Leukocytopenia	1 (16.7)	1	1 (16.7)	1	0 (0)	0	2 (11.1)	2
Grade 1	1 (16.7)	1	0 (0)	0	0 (0)	0	1 (5.6)	1
Grade 2	0 (0)	0	1 (16.7)	1	0 (0)	0	1 (5.6)	1
Anemia	3 (50.0)	3	0 (0)	0	2 (33.3)	2	5 (27.8)	5
Grade 1	3 (50.0)	3	0 (0)	0	2 (33.3)	2	5 (27.8)	5

### Secondary endpoints

**Fig. 2:**
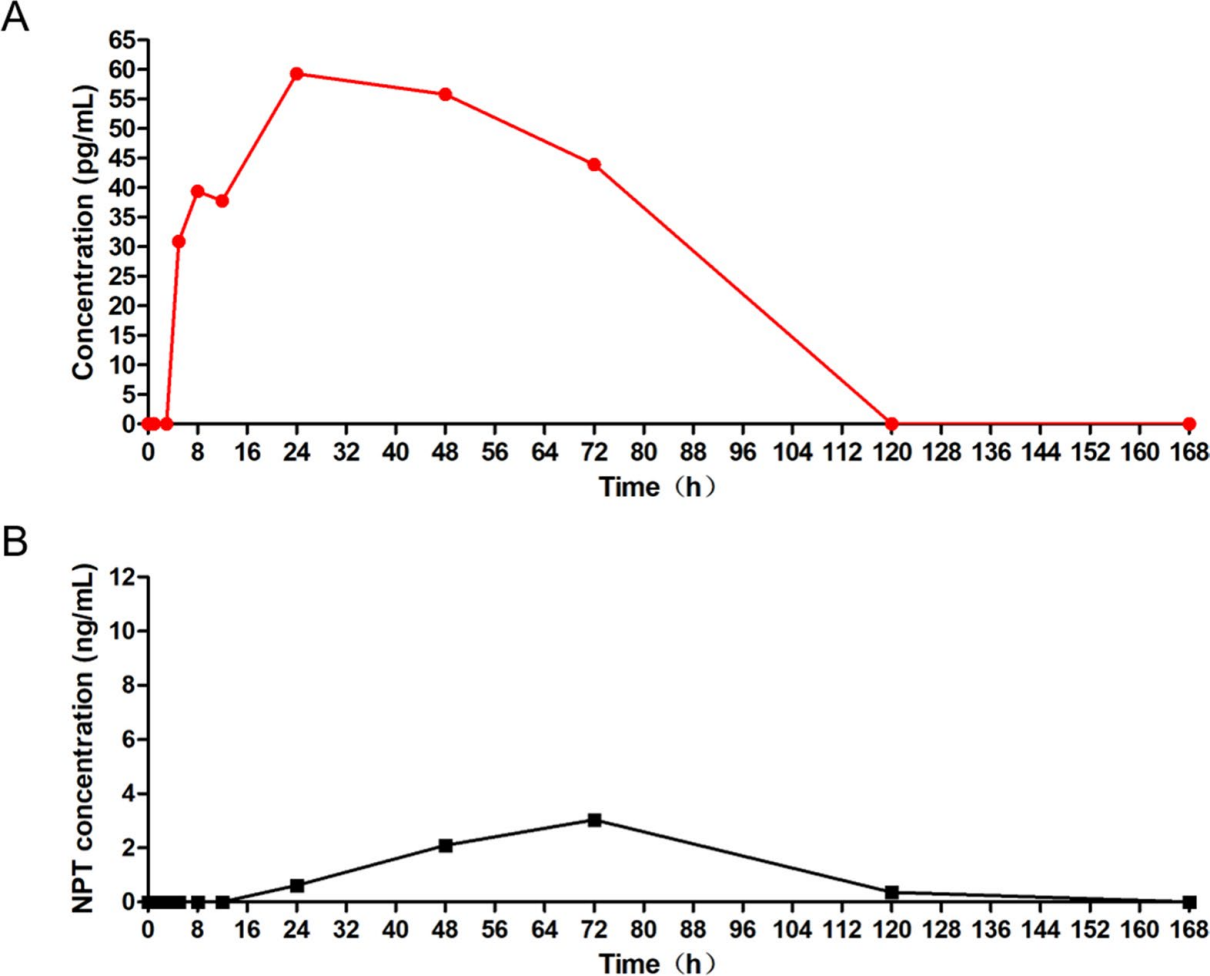
Pharmacokinetic and neopterin profiles of participant 005: (**A**) Pharmacokinetic characteristics, (**B**) Neopterin concentration. Abbreviation: NPT, neopterin

The concentration of PegIFNα-2b in participants was measured using a double-antibody sandwich ELISA method (linear range: 3.0 pg/mL to 120.0 pg/mL). The results showed that, with the exception of participant 005 in treatment group 2, all other participants had PegIFNα-2b concentrations below the lower limit of detection. Pharmacokinetic parameters could be calculated for participant 005: AUC_0 − t_ 3497.3 ng/Lh, AUC_0−∞_ 10821.59 ng/Lh, C_max_ 59.3 ng/L, T_max_ 24 h, t_1/2_ 72.66 h, CL 16.63 L/h, Vd 1743.87 L, MRT_0 − t_ 38.08 h, MRT_0−∞_ 87.52 h (Fig. 2A).

**Fig. 3:**
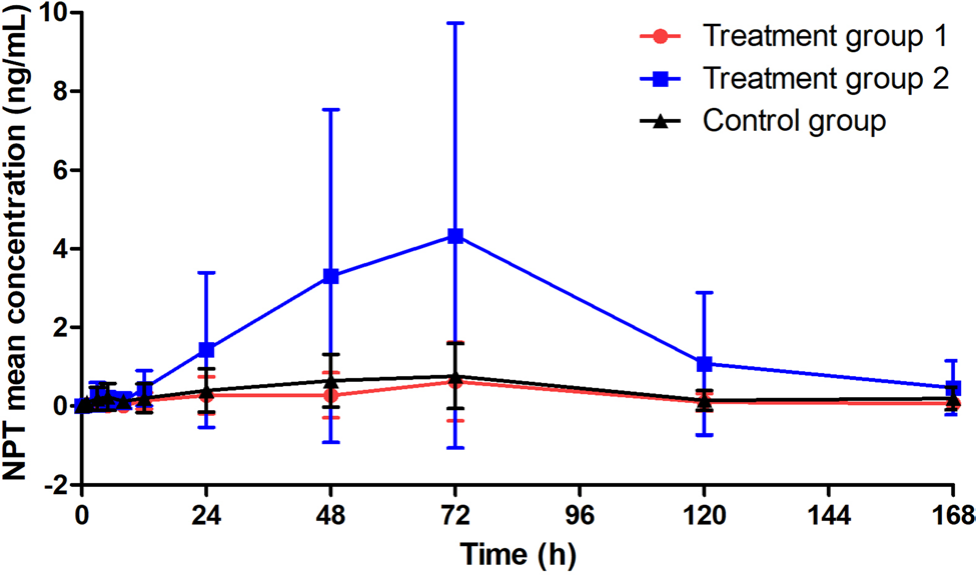
Curve of neopterin concentration over time (*n* = 18). Abbreviation: NPT, neopterin

The results (Table 3; Fig. 3) indicated that the neopterin levels in treatment group 1 and the control group were generally lower, while most participants in treatment group 2 showed slightly higher levels. However, no significant differences were observed among the three treatment groups (*P* > 0.05). Additionally, the neopterin level of participant 005 was comparable to that of the majority of subjects in treatment group 2 (Fig. 2B).

**Table 3:** Neopterin concentration (*n* = 18)

	Treatment group 1 (*n* = 6)	Treatment group 2 (*n* = 6)	Control group (*n* = 6)
C_max_ (ng/mL)	0.6183	4.333	0.7567
T_max_ (h)	72	72	72
AUC (h·ng/mL)	40.3	328.6	63.95
AUC (h·nmol/L)	159	1298	253

**Fig. 4:**
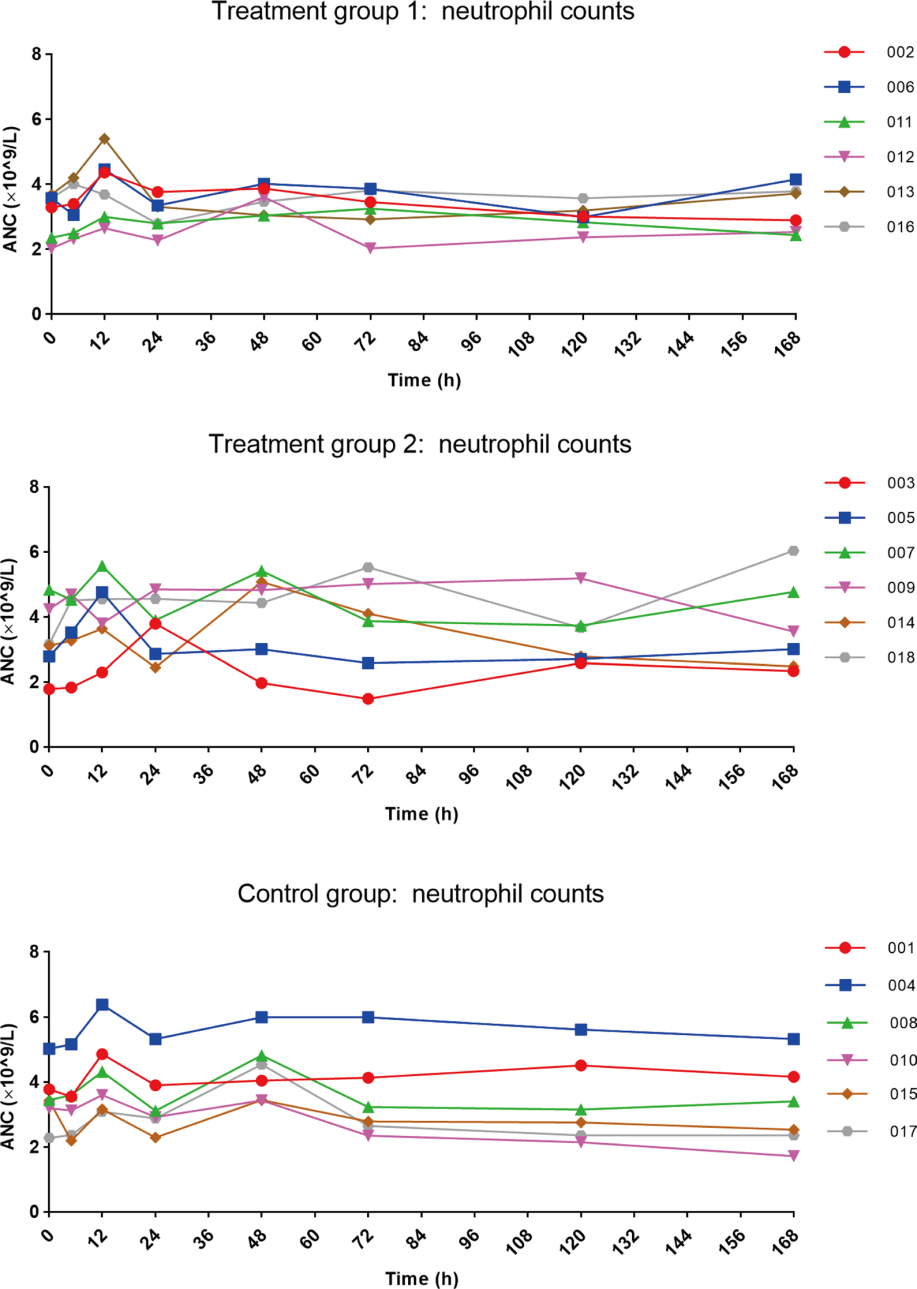
Trend of neutrophil counts in three groups (*n* = 18). Abbreviation: ANC, absolute neutrophil counts

After inhalation of nebulized PegIFNα-2b, there was a small peak in absolute neutrophil counts at 12 h post-dose. The average values for the three groups were: 3.927 × 10^9^ /L, 4.103 × 10^9^ /L, and 4.232 × 10^9^ /L (Fig. 4), all of which remained within the normal range with no statistically significant differences between the groups (*P* > 0.05). Within 24 h, the neutrophil counts of almost all participants returned to baseline levels.

**Fig. 5:**
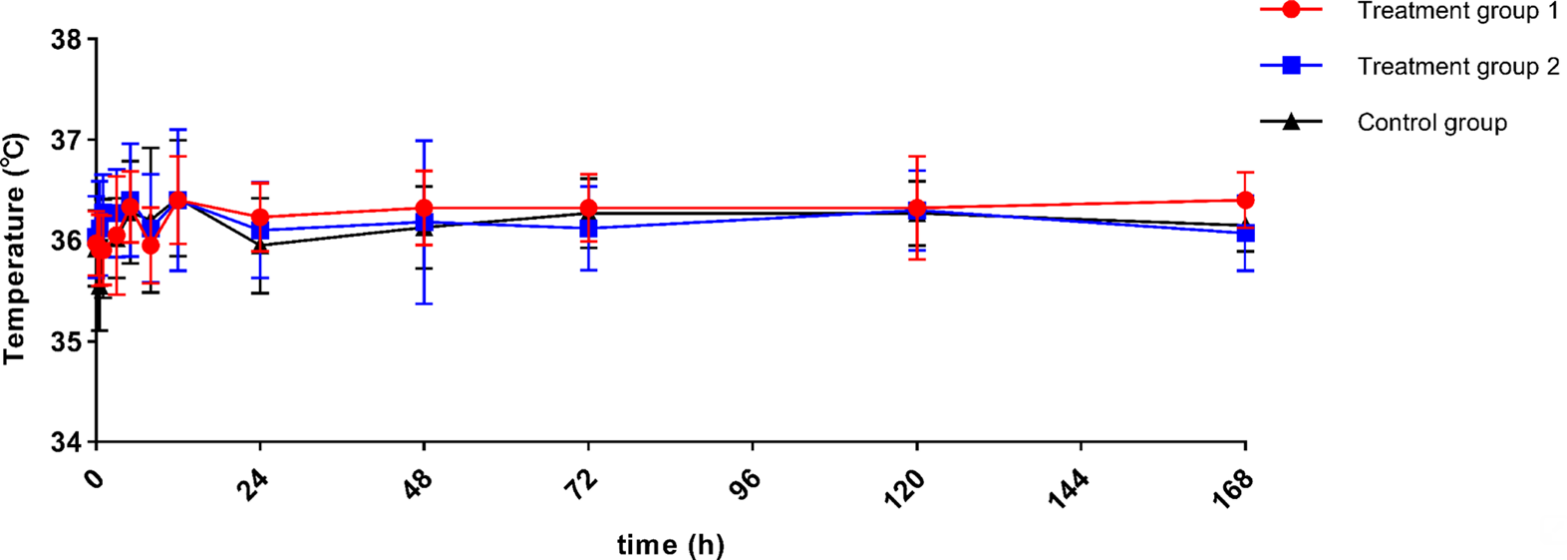
Trends of participants’ body temperature (*n* = 18)

There was no significant impact on the body temperature after inhalation of PegIFNα-2b. The body temperature of participants in all groups fluctuated within the normal range post-dose (Fig. 5).

## Discussion

This phase 1 clinical study marked the first application of nebulized inhalation of PegIFNα in healthy adults, aimed at assessing safety, tolerability, and conducting pharmacokinetic/pharmacodynamic investigations. The findings indicated that nebulized inhalation of PegIFNα-2b (90–180 µg) demonstrated good safety and tolerability, with potential systemic drug absorption observed.

Most subjects (17/18) had PegIFNα-2b concentrations in their blood below the limit of detection after nebulization, except for trace amounts detected in participant 005. Its C_max_ was approximately 2% for a subcutaneous injection of 90 µg and 0.6% for a subcutaneous injection of 180 µg, and AUC was approximately 1% for a subcutaneous injection of 90 µg and 0.3% for a subcutaneous injection of 180 µg [15]. Notably, participant 005 did not experience any adverse events, indicating that the trace systemic drug concentration did not pose additional safety risks. In a small-sample study evaluating the pharmacokinetics of nebulized inhalation of conventional IFNα [16], 3 out of 114 blood samples (2.6%) tested positive for IFNα after a single inhalation dose of 18 to 216 × 10^6^ IU, suggesting that both PegIFNα-2b and conventional IFNα may lead to systemic drug absorption after nebulization. However, the amount of drug absorbed into the bloodstream was minimal, which may explain the good tolerability observed in previous studies of nebulized inhalation of conventional IFNα for respiratory tract infections [17–18]. In this study, AEs were limited to anemia and leukopenia, with severity ranging from grade 1 to grade 2, requiring no additional intervention as they resolved spontaneously. The route of administration plays a significant role in determining the extent of systemic absorption and, consequently, the potential for AEs. For instance, a randomized controlled trial (RCT) exploring nebulized IFNα for the treatment of non-influenza viral pneumonia demonstrated that nebulized administration significantly reduced IFNα blood concentrations in normal tissues, which was associated with a reduction in AEs, with only 6.5% of participants experiencing any side effects [6]. Similarly, a study assessing the safety of nebulized IFNα in healthy subjects and patients with chronic bronchitis found that nebulized IFNα was well-tolerated, without significant vital sign alterations [17]. In contrast, systemic administration of IFNα through subcutaneous or intramuscular injection is associated with a high incidence of AEs, with overall AEs exceeding 90% and SAEs occurring in more than 15% of patients [19]. Previous studies suggeste that nebulized IFNα therapy activates local immune responses in the bronchoalveolar cells of the respiratory tract, targeting antiviral effects directly to the lungs without causing significant systemic toxicity. Any AEs that occur are typically mild and are likely inactivated during the transalveolar passage of the drug [17, 20]. Therefore, changing the administration of PegIFNα-2b from systemic to nebulized local delivery may help in minimizing systemic AEs and making it more acceptable to patients, especially for children who are susceptible to viral respiratory diseases.

In the context of immune response activation, neutrophils, macrophages, and dendritic cells secrete a type of cytokine called neopterin under the stimulation of interferon-γ, which serves as a novel marker for immune response in the body [21–23]. Neopterin production is specifically linked to the activation of the IFN-γ receptor signaling pathway, which is central to the biological activity of IFN-based therapies, including PegIFNα-2b. Furthermore, a clear dose-response relationship has been observed between neopterin levels and IFNα dosage. A preliminary study demonstrated that the AUC of neopterin increased with PegIFNα-2b doses ranging from 45 to 270 µg, reaching a plateau at 180 µg, suggesting receptor saturation effects [15]. This dose-response relationship highlights neopterin as a sensitive marker for evaluating the biological activity of PegIFNα-2b. Additionally, neopterin’s dynamics have been validated through mechanism-based pharmacokinetic-pharmacodynamic models, which accurately reflect its interaction with IFNα receptor binding and downstream signaling processes [24]. In this study, the concentrations of neopterin in the blood were detectable in all subjects after inhalation of PegIFNα-2b, indicating the possibility of drug entry into the bloodstream. Although both treatment group 2 and the control group inhaled PegIFNα-2b at a dosage of 180 µg, their overall performances were somewhat inconsistent (with AUCs of 328.6 h·ng/mL and 63.95 h·ng/mL, respectively), whereas the control group demonstrated a similarity to treatment group 1 (nebulized PegIFNα-2b 90 µg) (with AUCs of 63.95 h·ng/mL and 40.3 h·ng/mL, respectively), suggesting a potential influence of co-administration with Ambroxol Hydrochloride. While there are no existing studies on the interaction between Ambroxol Hydrochloride and PegIFNα-2b, we speculated on potential mechanisms: First, Ambroxol Hydrochloride’s instability under certain conditions (e.g., temperature, pH shifts, elevated humidity) may degrade PegIFNα-2b through reactive intermediate generation, compromising its stability and efficacy [25–26]. Second, as a mucolytic agent, Ambroxol Hydrochloride reduces mucus viscosity and enhances mucociliary clearance, which may accelerate the removal of PegIFNα-2b from the respiratory epithelium, thereby shortening its absorption window [25]. We hypothesize that the mucus-modifying effects of Ambroxol Hydrochloride may reduce the residence time of PegIFNα-2b at the respiratory epithelium, thereby leading to diminished contact duration with target cells. Future studies could validate this hypothesis through comparative measurements of drug concentrations in bronchoalveolar lavage fluid between monotherapy and combination therapy groups using preclinical animal models. Third, Ambroxol Hydrochloride may compete with PegIFNα-2b for binding sites or directly interfere with its biological activity, as suggested by computational studies [27]. Therefore, further investigation into the combined effects of different drugs administered via inhalation is needed in subsequent studies. While the concentrations of neopterin were positively correlated with the dosage of PegIFNα-2b in both treatment group 1 and treatment group 2, there was no statistically significant difference (*P* > 0.05), possibly due to the sample size. Additionally, compared with previous pharmacokinetic studies of PegIFNα-2b (Figure S1), the AUCs of neopterin after inhalation of PegIFNα-2b in the three groups were 5%, 40%, and 8% of the respective subcutaneous injection doses. This further underscored the dose-dependency of the pharmacological effects of inhaled PegIFNα-2b, as well as the significantly lower blood exposure observed with nebulized administration compared to systemic delivery at the same dose. Currently, there is limited research exploring the relationship between different nebulized doses of IFNα and clinical efficacy. Therefore, in the next phase of our research, we aim to further investigate the dose-response relationship in patients with lower respiratory tract infections to identify the optimal dosing for therapeutic effectiveness.

Changes in neutrophil counts and body temperature are among the most common laboratory and clinical responses following systemic IFNα administration, reflecting early immune activation and serving as sensitive pharmacodynamic markers [28–31]. Therefore, in this study, neutrophil counts and body temperature were included as additional markers to provide a more comprehensive assessment of the biological effects following PegIFNα-2b administration. For most subjects, neutrophils reached a minor peak at 12 h post-administration, as evidenced by enhanced signals in blood drug concentration (Figure S2). Although the detected absorbance (OD values) was very low, indicating rapid entry of minute amounts of PegIFNα-2b into the bloodstream shortly after administration, peak neutrophil levels returned to baseline within 24 h and remained within the normal range at all subsequent measurement points. Overall, the hematological response following inhalation of PegIFNα-2b was minimal. While neopterin is a marker for immune activation, the data did not reveal a significant correlation between neopterin levels and clinical markers such as neutrophil counts. The delayed peak in neopterin concentrations (72 h) compared to the transient changes in neutrophil counts (12 h) suggested that neopterin may reflect a later phase of the immune response in this context. Moreover, the body temperature of participants in all groups remained within the normal range.

Recent studies highlighted the complex roles of inflammatory proteins in disease modulation. For instance, Zheng et al. revealed that cytokines like IL-6 exert dual effects depending on their localization, a phenomenon paralleled by our observation of minimal systemic PegIFNα-2b absorption despite local action [32–33]. Furthermore, the dose-dependent inflammatory modulation reported in spinal degeneration supports our exploration of higher PegIFNα-2b doses in future trials to enhance efficacy without compromising safety [34].

This study has several limitations. First, the sample size was relatively small, with only 18 participants, which limited the statistical power and generalizability of the findings. Given that there are no prior studies investigating the nebulized administration of PegIFNα in humans, we adopted a cautious approach by enrolling a limited number of participants in this early exploratory stage. Since the primary aim of this study was to assess the safety, tolerability, and pharmacokinetics/pharmacodynamic of nebulized PegIFNα-2b, rather than its efficacy, a formal power analysis was not conducted. Based on the results of this initial investigation, future studies will need to include larger cohorts to better assess efficacy and further validate the safety and pharmacokinetic profile of nebulized PegIFNα-2b. Second, this study did not measure the concentration of PegIFNα-2b in airway secretions, leaving important questions about its targeted delivery to the lungs and its potential antiviral effects unanswered. This limitation is particularly significant because several factors may affect local drug delivery to the lungs, including physical barriers in the respiratory tract, mucociliary clearance mechanisms, and airflow dynamics [35–37]. Furthermore, airflow interference during nebulization could lead to uneven drug deposition, limiting effective delivery to the target area. Since the current study focused on systemic pharmacokinetics through blood samples, this gap in data highlights a crucial area for future research. We plan to address this limitation in subsequent studies by measuring PegIFNα-2b concentrations in airway secretions and investigating its local pharmacokinetics and antiviral effects within the respiratory system. Moreover, while this study suggested a potential pharmacodynamic interaction between PegIFNα-2b and Ambroxol Hydrochloride, the mechanistic basis for this interaction remains speculative. We have discussed several possible mechanisms, but further experimental data are required to confirm these hypotheses. Finally, the 7-day follow-up was based on unpublished animal data suggesting that nebulized PegIFNα-2b remained in the lungs for about 72 h. Thus, we focused on pharmacokinetic, pharmacodynamic and safety within this timeframe. Future studies with extended follow-up durations are needed to explore these aspects more thoroughly.

## Conclusions

This preliminary study suggested that nebulized PegIFNα-2b at doses of 90 µg and 180 µg appeared to be well-tolerated and showed acceptable safety in healthy adults. Trace amounts of PegIFNα-2b were detected in the bloodstream following inhalation, though the associated hematological changes were minimal. Given the exploratory nature of this study, further investigation with larger sample sizes is needed to better understand the safety, pharmacokinetics, and potential therapeutic effects of inhaled PegIFNα-2b, particularly in patients with lower respiratory tract infections.

## Electronic supplementary material

Below is the link to the electronic supplementary material.

Supplementary Material 1

## Data Availability

No datasets were generated or analysed during the current study.

## References

[1] Mizgerd JP. Acute lower respiratory tract infection. N Engl J Med. 2008;358:716–27. 18272895 10.1056/NEJMra074111 PMC2711392

[2] Yang L, Zhang G, Huang L, et al. The effect of Recombinant human interferon α1b treatment of infants hospitalized with lower respiratory tract infection on subsequent wheezing. J Pediatr (Rio J). 2021;97:617–22. 33592175 10.1016/j.jped.2020.12.005 PMC9432140

[3] Ji L, Li T, Chen H, et al. The crucial regulatory role of type I interferon in inflammatory diseases. Cell Biosci. 2023;13:230. 38124132 10.1186/s13578-023-01188-z PMC10734085

[4] Zhao D, Liu H, Liu F. Efficacy and safety of nebulized Recombinant human interferon α2b in the treatment of infantile bronchiolitis: a randomized controlled multicenter study. Chin J Practical Pediatr. 2016;31:1095–100.

[5] Sun H, Zhu A, Guo Y. Efficacy analysis of nebulized Recombinant human interferon α2b combined with low-dose Methylprednisolone in the treatment of severe viral pneumonia in children. China Med Guide. 2019;21:5.

[6] Jiang R, Han B, Song M, et al. Efficacy and safety of aerosol inhalation of Recombinant human interferon α1b (IFNα1b) injection for noninfluenza viral pneumonia, a multicenter, randomized, double-blind, placebo-controlled trial. J Inflamm (Lond). 2020;17:19. 32431566 10.1186/s12950-020-00249-1 PMC7221328

[7] Qin L, Cui Z, Wu Y, et al. Challenges and strategies to enhance the systemic absorption of inhaled peptides and proteins. Pharm Res. 2023;40:1037–55. 36385216 10.1007/s11095-022-03435-3 PMC9668393

[8] Swierczewska M, Lee KC, Lee S. What is the future of pegylated therapies? Expert Opin Emerg Drugs. 2015;20:531–6. 26583759 10.1517/14728214.2015.1113254 PMC4908577

[9] McLeod VM, Chan LJ, Ryan GM, et al. Optimal pegylation can improve the exposure of interferon in the lungs following pulmonary administration. J Pharm Sci. 2015;104:1421–30. 25631360 10.1002/jps.24353

[10] Zhao H, Kurbanov F, Wan M, et al. Genotype B and younger patient age associated with better response to low-dose therapy: a trial with pegylated/nonpegylated interferon-alpha-2b for hepatitis B e antigen-positive patients with chronic hepatitis B in China. Clin Infect Dis. 2007;44:541–8. 17243057 10.1086/511042

[11] Li W, Wang M, Kong L, et al. Peginterferon alpha-based therapy for chronic hepatitis B focusing on HBsAg clearance or seroconversion: a meta-analysis of controlled clinical trials. BMC Infect Dis. 2011;11:165. 21651820 10.1186/1471-2334-11-165 PMC3128052

[12] Hou F, Yin Y, Zeng L. Efficacy and safety analysis of polyethylene glycol interferon α-2b (Y type, 40kD) injection in patients with HBeAg-positive chronic hepatitis B. Chin J Hepatol. 2017;25:589–96.

[13] Shen K, Hong J, Yu G. Guidelines for standardized management of nebulization centers for children. Volume 22. Beijing: People’s Medical Publishing House; 2014.

[14] Li H, Ji C, Zong X. Standardized growth curves for height and weight of Chinese children and adolescents aged 0–18 years. Chin J Pediatr. 2009;47:487–92. 19951507

[15] Wang R, Qi W, Zhao Q. Pharmacokinetics and pharmacodynamics of polyethylene glycol-modified Recombinant human interferon α2b injection (Y type) in healthy human body. Chin J Clin Pharmacol. 2011;27:395–7.

[16] Sherling DH, Perumareddi P, Hennekens CH. Metabolic syndrome. J Cardiovasc Pharmacol Ther. 2017;22:365–7. 28587579 10.1177/1074248416686187

[17] Giosuè S, Casarini M, Ameglio F, et al. Minimal dose of aerosolized interferon-alpha in human subjects: biological consequences and side-effects. Eur Respir J. 1996;9:42–6. 8834332 10.1183/09031936.96.09010042

[18] Yu J, Lu X, Tong L, et al. Interferon-α-2b aerosol inhalation is associated with improved clinical outcomes in patients with coronavirus disease-2019. Br J Clin Pharmacol. 2021;87:4737–46. 33982806 10.1111/bcp.14898 PMC8239515

[19] Ma M, Qian J, Fu L, et al. Comparative study on adverse reactions of Recombinant human interferon α2a and α2b injections. Chin J Mod Appl Pharm. 2023;40(5):677–82.

[20] Bocci V, Pessina GP, Paccini A, et al. Pulmonary catabolism of interferons: alveolar absorption of ^125^I-labelled human interferonalpha is accompanied by partial loss of biological activity. Antiviral Res. 1984;4:211–22. 6486767 10.1016/0166-3542(84)90019-6

[21] Bitterlich G, Szabó G, Werner ER, et al. Selective induction of mononuclear phagocytes to produce neopterin by interferons. Immunobiology. 1988;176:228–35. 2452128 10.1016/S0171-2985(88)80055-X

[22] Murr C, Widner B, Wirleitner B, et al. Neopterin as a marker for immune system activation. Curr Drug Metab. 2002;3:175–87. 12003349 10.2174/1389200024605082

[23] Jeon S, Juhn JH, Han S, et al. Saturable human neopterin response to interferon-α assessed by a pharmacokinetic-pharmacodynamic model. J Transl Med. 2013;11:240. 24088361 10.1186/1479-5876-11-240 PMC3853247

[24] Mager DE, Neuteboom B, Efthymiopoulos C, et al. Receptor-mediated pharmacokinetics and pharmacodynamics of interferon-beta1a in monkeys. J Pharmacol Exp Ther. 2003;306(1):262–70. 12660309 10.1124/jpet.103.049502

[25] Jelić D, Papović S, Vraneš M, et al. Thermo-analytical and compatibility study with mechanistic explanation of degradation kinetics of ambroxol hydrochloride tablets under non-isothermal conditions. Pharmaceutics. 2021;13(11):1910. 10.3390/pharmaceutics13111910 PMC8621728 34834325

[26] Wang M. Studies on the effect of pH buffer solutions with different concentrations on the stability of ambroxol hydrochloride injection. MRP. 2024;2(8):18–20.

[27] Kehinde IA, Egbejimi A, Kaur M, et al. Inhibitory mechanism of ambroxol and bromhexine hydrochlorides as potent blockers of molecular interaction between sars-cov-2 Spike protein and human angiotensin-converting enzyme-2. J Mol Graph Model. 2022;114:108201. 35487151 10.1016/j.jmgm.2022.108201 PMC9022787

[28] Marcellin P, Lau GKK, Bonino F, et al. Peginterferon Alfa-2a alone, lamivudine alone, and the two in combination in patients with HBeAg-Negative chronic hepatitis B. N Engl J Med. 2004;351(12):1206–17. 15371578 10.1056/NEJMoa040431

[29] García-García I, Hernández-González I, Díaz-Machado A, et al. Pharmacokinetic and pharmacodynamic characterization of a novel formulation containing co-formulated interferons alpha-2b and gamma in healthy male volunteers. BMC Pharmacol Toxicol. 2016;17(1):58. 27923408 10.1186/s40360-016-0103-8 PMC5142133

[30] Expert Committee on Clinical Management of Adverse Reactions in Interferon-α Therapy for Chronic Viral Hepatitis. Expert consensus on clinical management of adverse reactions in interferon-α therapy for chronic viral hepatitis. J Clin Hepatol. 2014;30(11):1106–11.

[31] Benguigui M, Cooper TJ, Kalkar P, et al. Interferon-stimulated neutrophils as a predictor of immunotherapy response. Cancer Cell. 2024;42(2):253–e26512. 38181798 10.1016/j.ccell.2023.12.005 PMC10864002

[32] Zheng Q, Lin R, Wang D, et al. Effects of Circulating inflammatory proteins on spinal degenerative diseases: evidence from genetic correlations and Mendelian randomization study. JOR Spine. 2024;7(2):e1346. 38895179 10.1002/jsp2.1346 PMC11183170

[33] Zheng Q, Wang D, Lin R, et al. Mendelian randomization analysis suggests no associations of human herpes viruses with amyotrophic lateral sclerosis. Front NeuroSci. 2023;17:1299122. 38156274 10.3389/fnins.2023.1299122 PMC10754516

[34] Zheng Q, Wang D, Lin R, et al. Effects of Circulating inflammatory proteins on osteoporosis and fractures: evidence from genetic correlation and Mendelian randomization study. Front Endocrinol. 2024;15:1386556. 10.3389/fendo.2024.1386556 PMC11097655 38757000

[35] Ma Y, Zhao J. Can inhalation corticosteroids substitute for systemic hormones therapy in airflow restricted diseases: questions and answers. Chin J Gen Practitioners. 2017;16(11):839–42.

[36] Labiris NR, Dolovich MB. Pulmonary drug delivery. Part I: physiological factors affecting therapeutic effectiveness of aerosolized medications. Br J Clin Pharmacol. 2003;56(6):588–99. 14616418 10.1046/j.1365-2125.2003.01892.x PMC1884307

[37] Ari A, Atalay OT, Harwood R, et al. Influence of nebulizer type, position, and bias flow on aerosol drug delivery in simulated pediatric and adult lung models during mechanical ventilation. Respir Care. 2010;55(7):845–51. 20587095

