# Supplementary material for: The safety, tolerability, pharmacokinetics, and pharmacodynamics of nebulized pegylated interferon α-2b in healthy adults: a randomized phase 1 trial
Source: BMC Pharmacol Toxicol. 2025 May 12;26:99. doi: 10.1186/s40360-025-00937-9 (PMC12070635; doi:10.1186/s40360-025-00937-9)
Supplement: Supplementary file 1 — Supplementary Material 1 [file 40360_2025_937_MOESM1_ESM.docx]

**SUPPLEMENTARY MATERIAL**


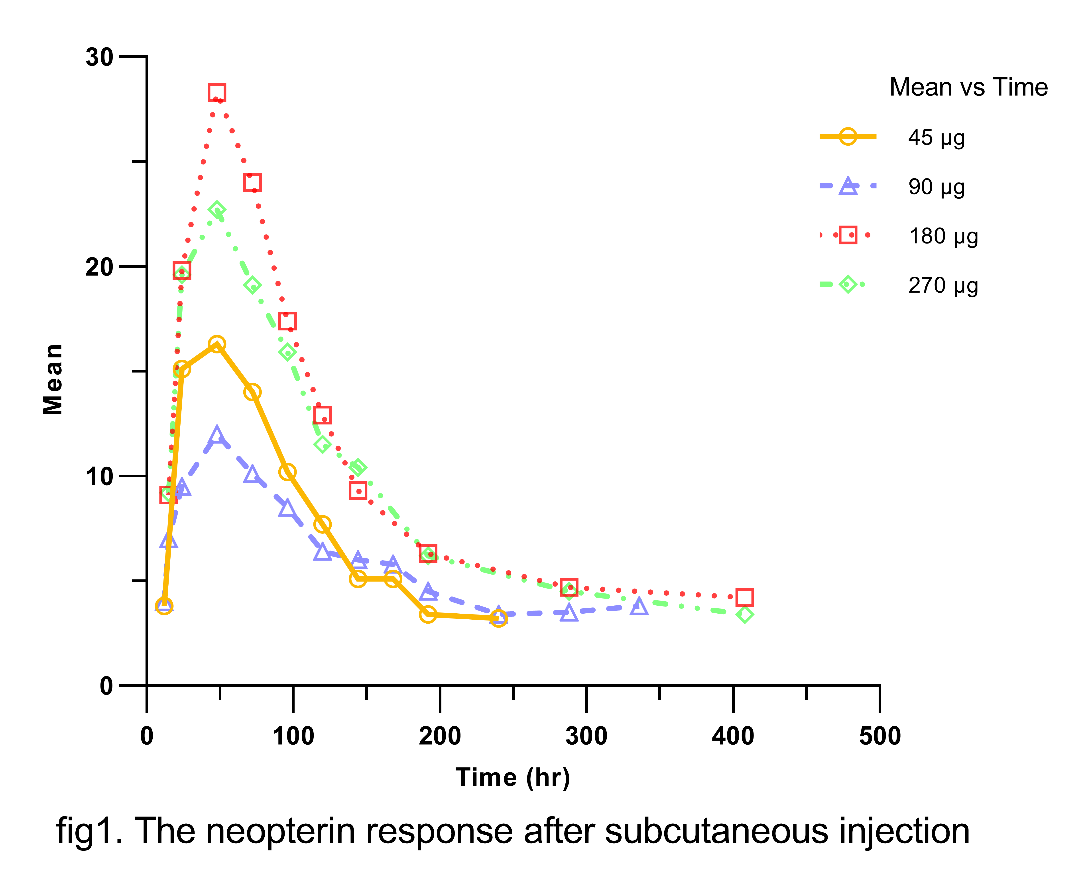


|  | **45μg** | **90μg** | **180μg** |
| --- | --- | --- | --- |
| **C_max_（nmol/L）** | **16.4** | **12.3** | **28.34** |
| **AUC（h·nmol/L）** | **1802** | **1468** | **3245** |

**Figure S1. The levels of neopterin following subcutaneous administration of PegIFNα-2b**


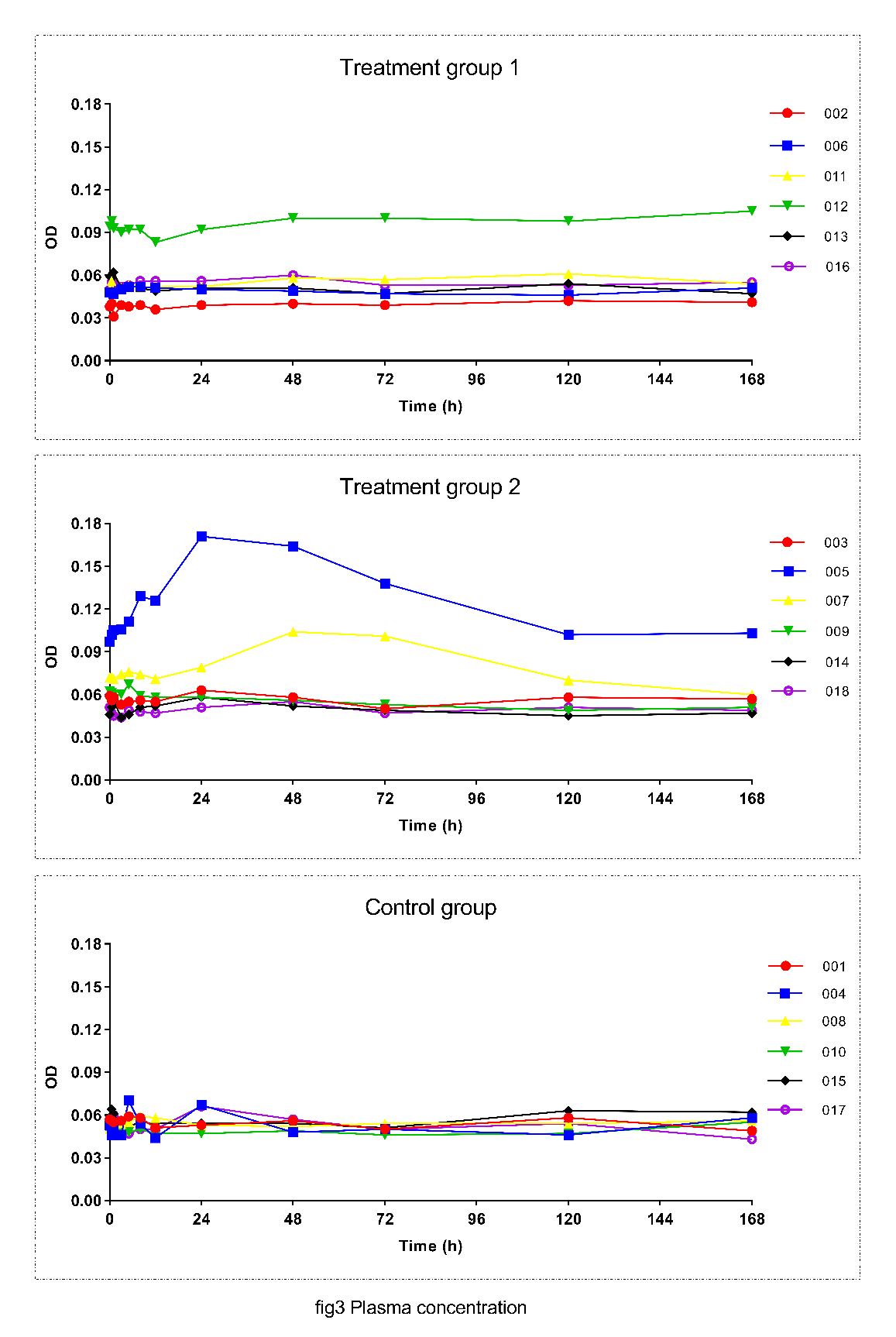


**Figure S2. The absorbance values of PegIFNα-2b in the blood samples of each participant.**
